# *Sapovirus* Gastroenteritis in Young Children Presenting as Distal Small Bowel Obstruction: A Report of 2 Cases and Literature Review

**DOI:** 10.1155/2016/6302875

**Published:** 2016-11-06

**Authors:** Lynn Model, Cathy Anne Burnweit

**Affiliations:** Nicklaus Children's Hospital, Miami, FL, USA

## Abstract

Abdominal pain and distention in children are commonly encountered problems in the pediatric emergency room. The majority of complaints are found to be due to benign entities such as gastroenteritis and constipation. What confounds these diagnoses is that young children often deliver a challenging and unreliable exam. Thus, it often becomes exceedingly problematic to differentiate these benign conditions from surgical conditions requiring prompt attention including small or large bowel obstruction, volvulus, and appendicitis. The cases highlight* Sapovirus* as a cause of severe abdominal distention and vomiting in children and this report is the first to describe and demonstrate the impressive radiologic findings that may be associated with this infection. Surgeons should heed this information and hesitate to emergently operate on similar children.

## 1. Introduction

Severe abdominal distention in young children, with symptoms and X-rays consistent with bowel obstruction, causes significant concern for physicians and families. An English language literature review revealed that* Sapovirus* as a cause of this clinical picture has not been described. In this report, we outline the courses of 2 children presenting with what appeared to be small bowel obstruction but who, after* Sapovirus* infectious gastroenteritis was diagnosed, went on to resolve their illnesses spontaneously.

## 2. Case 1

A 2-year-old male with no significant past medical history and normal stooling history presented to the emergency room (ER) with nonbilious vomiting and severe abdominal distention for one week. He was initially seen at an urgent care center where he was given ondansetron intramuscularly and discharged home. He had slight improvement and then subsequent worsening of the vomiting. During the 2 days prior to presentation in the ER, the patient had been irritable, fussy, and less active than normal. He was also reported to have anorexia and diarrhea containing “red flakes.” The patient's mother denied urinary symptoms, upper respiratory symptoms, or recent travel in the boy but noted a sibling with gastroenteritis approximately 3 weeks prior.

**Figure 1:**
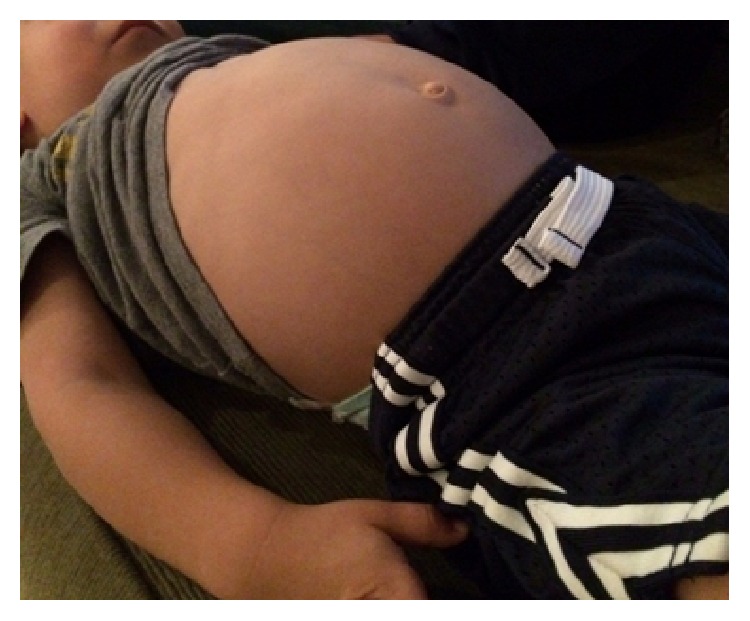
Photograph of severe abdominal distention in the 2-year-old male presented in Case 1.

**Figure 2:**
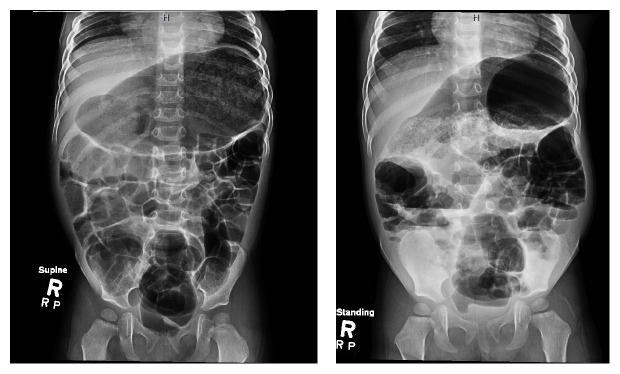
Supine and upright plain X-rays of the patient presented in Case 1. Moderate dilatation of the bowel is seen with significant gaseous distention of the stomach being seen. Multiple air-fluid levels are seen. No free intra-abdominal air is identified.

Exam revealed a markedly distended, tympanitic abdomen without significant tenderness (Figure 1). Plain films demonstrated markedly dilated loops of bowel with air-fluid levels (Figure 2); distal bowel obstruction was suspected. Ultrasound was negative for intussusception. Laboratory studies revealed a slight leukocytosis at 11,500/mL with a monocytosis of 17.8% and low neutrophils at 19.7%. Electrolytes and liver functions were all within normal ranges. Stool for culture and polymerase chain reaction (PCR) was sent. Fecal hemoccult test was negative.

The patient was admitted for hydration and control of nausea with ondansetron. A nasogastric tube was placed but was promptly removed by the patient repeatedly. Abdominal distention resolved over the next 24 hours; the patient was fed a regular diet and discharged home. Stool PCR returned positive for* Sapovirus*, and neither PCR nor culture revealed other pathogens.

## 3. Case 2

A 2-year-old male with a history of mild prematurity (35 weeks of gestational age), laryngomalacia, and vocal cord paralysis with tracheostomy presented to the ER with severe abdominal distention and pain. The boy's mother described one week of nonbloody diarrhea and then no bowel movement in the prior 3 days and prior to that no problems with stooling. The patient had a history of gastrostomy and fundoplication as an infant but had been maintaining nutrition by oral feeding for the past 7 months. He was not vomiting but was brought in due to the concern for his massive abdominal girth.

**Figure 3:**
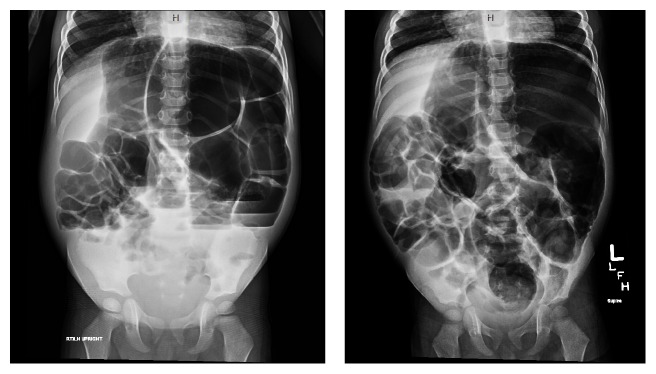
Supine and upright plain X-rays of the patient presented in Case 2. The stomach is markedly distended and filled with air. There is moderate and severe distention of several bowel loops, with air-fluid levels.

Exam revealed a distended, nontender, tympanitic abdomen and no other abnormalities. X-ray showed markedly dilated loops of bowel with air-fluid levels (Figure 3), and small bowel obstruction was the lead diagnosis, given his surgical history. Laboratory exams revealed normal electrolytes, liver function tests, and white cell count (9,100/mL with normal differentiation).

The patient was admitted for observation, with hydration and withholding of oral intake. Shortly after admission, he began passing profuse amounts of flatus. His distention significantly improved. On hospital day 2, he had a stool, and feeding was begun. The fecal PCR returned positive for* Sapovirus* and no other pathogens. The patient recovered quickly and was discharged home.

## 4. Discussion

The differential diagnosis of suspected bowel obstruction in young children is vast. It includes such entities as postoperative adhesions, intussusception, Meckel's diverticulum, appendicitis, and foreign body ingestion, all of which may require prompt surgical intervention. Other nonsurgical etiologies are gastroenteritis, constipation, medication-related side effects, and enteric neuropathies, such as Hirschsprung's disease or pseudoobstruction syndromes [1, 2]. To complicate matters, young children often deliver a difficult and unreliable exam.

*Sapovirus* is a member of the Caliciviridae family that worldwide is responsible for 2.2–12.7% of gastroenteritis and has been reported in more than 35 countries [3–5]. Incubation period is from 1 to 4 days, and there is a median of 6 days of symptoms. Major symptoms include diarrhea and vomiting, as well as other common viral symptoms such as nausea, abdominal pain, headaches, myalgia, and malaise. Fever is rare, and the symptoms are usually self-limited [5]. This entity more commonly affects children under 5 years of age than in older children and adults, though outbreaks in hospitalized adult populations have been described [6]. Asymptomatic viral shedding has been reported as well.* Sapovirus* has been studied significantly less than other more common Caliciviridae like norovirus. The clinical presentation of nonbloody diarrhea and vomiting is generally milder than seen with rotavirus or norovirus, but hospitalizations and deaths have been reported [5], particularly in rural areas without running water and in immunocompromised children [3]. PCR assays are the best current method for detection of viral strains [7].* Sapovirus* is often detected concomitantly with other viruses such as* norovirus*,* rotavirus*,* adenovirus*, and human* astrovirus* in children with gastroenteritis [8].

The pathophysiology of* Sapovirus* is not well-studied but it is believed to act similar to* rotavirus* and other Caliciviridae such as* norovirus*. For example,* rotavirus* causes infectious diarrhea through production of enterotoxins that alter the epithelial cell function and permeability and through activation of the enteric nervous system.* Norovirus* similarly damages the villi of the small bowel and causes diminished activity of intestinal disaccharidases leading to malabsorption [9]. Effects on the enteric nervous system along with increased luminal contents from malabsorption may thus explain the bowel dilation seen in the presented cases.

We have presented a report of 2 children with exams and imaging greatly concerning for bowel obstruction but who were found to have gastroenteritis with* Sapovirus* as the causative organism. Thus, the approach to a child with abdominal distention, even those with significant air-fluid levels on X-ray and profuse vomiting, should be to consider a wide differential diagnosis. Surgical consultation should be called when there is concern for mechanical obstruction. Clinicians should recognize that even when patients have worrisome symptoms and X-rays, if they lack findings of an acute abdomen, it may be prudent to postpone invasive testing or surgical intervention for a period of observation, while stool cultures and PCR results are evaluated.

## References

[1] Ambartsumyan L., Rodriguez L. (2014). Gastrointestinal motility disorders in children. *Gastroenterology & Hepatology*.

[2] Gfroerer S., Rolle U. (2015). Pediatric intestinal motility disorders. *World Journal of Gastroenterology*.

[3] Page N., Groome M. J., Murray T. (2016). Sapovirus prevalence in children less than five years of age hospitalised for diarrhoeal disease in South Africa, 2009–2013. *Journal of Clinical Virology*.

[4] Liu X., Yamamoto D., Saito M. (2015). Molecular detection and characterization of sapovirus in hospitalized children with acute gastroenteritis in the Philippines. *Journal of Clinical Virology*.

[5] Oka T., Wang Q., Katayama K., Saif L. J. (2015). Comprehensive review of human sapoviruses. *Clinical Microbiology Reviews*.

[6] Svraka S., Vennema H., van Der Veer B. (2010). Epidemiology and genotype analysis of emerging sapovirus-associated infections across Europe. *Journal of Clinical Microbiology*.

[7] Osborne C. M., Montano A. C., Robinson C. C., Schultz-Cherry S., Dominguez S. R. (2015). Viral gastroenteritis in children in Colorado 2006–2009. *Journal of Medical Virology*.

[8] Thongprachum A., Khamrin P., Maneekarn N., Hayakawa S., Ushijima H. (2016). Epidemiology of gastroenteritis viruses in Japan: prevalence, seasonality, and outbreak. *Journal of Medical Virology*.

[9] Navaneethan U., Giannella R. A. (2008). Mechanisms of infectious diarrhea. *Nature Clinical Practice Gastroenterology and Hepatology*.

